# The emergence of temporality in attitudes towards cryo-fertility: a case study comparing German and Israeli social egg freezing users

**DOI:** 10.1007/s40656-022-00495-x

**Published:** 2022-05-17

**Authors:** Nitzan Rimon-Zarfaty, Silke Schicktanz

**Affiliations:** 1 Department of Medical Ethics and History of Medicine, University Medical Center Göttingen, Göttingen, Lower Saxony Germany; 2 Department of Human Resource Management Studies, Sapir Academic College, D.N. Hof Ashkelon 7916500, Hof Ashkelon, Israel

**Keywords:** Cryo-fertility, Cryopolitics, Temporality, Social egg freezing, Germany, Israel

## Abstract

Assistive reproductive technologies are increasingly used to control the biology of fertility and its temporality. Combining historical, theoretical, and socio-empirical insights, this paper aims at expanding our understanding of the way temporality emerges and is negotiated in the contemporary practice of cryopreservation of reproductive materials. We first present an historical overview of the practice of cryo-fertility to indicate the co-production of technology and social constructions of temporality. We then apply a theoretical framework for analysing cryobiology and cryopreservation technologies as creating a new epistemic perspective interconnecting biology and temporality. Thereafter, we focus on the case of ‘social egg freezing’ (SEF) to present socio-empirical findings illustrating different reproductive temporalities and their connection to the social acceptance of and expectations towards the practice. SEF is a particularly interesting case as it aims to enable women to disconnect their reproductive potential from their biological rhythms. Based on 39 open interviews with Israeli and German SEF users, the cross-cultural comparative findings reveal three types of attitudes: postponing motherhood/reproductive decisions (German users); singlehood and “waiting” for a partner (Israeli and German users); and the planning of and hope for multiple children (Israeli users). For theory building, this analysis uncovers temporality formations embedded in gender and reproductive moral values; including the ‘extended present’, ‘waiting’, and ‘reproductive futurism’. We conclude by discussing the contribution of our findings by advancing the theoretical framework of ‘cryopolitics’ highlighting the theoretical implications and importance of gendered and cultural imaginaries (re)constructing medical technological innovations and related temporalities.

## Introduction

On March 2020, with the first outbreak of the COVID-19 pandemic leading to the first lockdown in Israel, Maayan Adam, an Israeli celebrity and television presenter, published a short column in “Israel Hayom” (a daily newspaper) in which she revealed her decision (after a year of deliberation and hesitation) to undertake the procedure of social egg freezing. In the article Adam writes: “That night, when they informed that Israel is closed due to war [in COVID-19]—I heard one thing only: “you are out of time sweetheart”. What you did not manage to do will no longer suffice in the near future. But learn for next time: [freeze your eggs] Now, (…) not later, not even in one minute” (Adam, 2020). This quotation, presented in a unique ‘eye opening’ context, highlights the decision and use of social egg freezing as embedded in perceptions of time and human temporality. First, it includes ideas regarding the time of procreational processes and their finality, highlighting dimensions of irreversibility. Second, it reflects ideals of timing and notions ‘right time’ for reproduction that rest on morally loaded understandings of the desirable course of individual lives leading to concerns that one is ‘not on time’. Third, it reflects a sense of urgency, of being ‘out of time’, of time as ‘fleeting’ or ‘ticking away’. Finally, it illustrates future orientation and expectations, also connected to ideas of responsibility and control.

Indeed, time plays a pivotal role when it comes to fertility and reproduction. Especially, since female fertility is characterized by a menstrual cycle and repetitive rhythm (Thompson, 2005) while pointing to a biologically limited time-frame, reflected in the common metaphor of the “biological-clock” (Amir, 2006). While assistive reproductive technologies were initially aimed at treating and overcoming biological infertility, such technologies are nowadays increasingly used to control also its temporal characteristics, aiming to expand the time-frame of fertility and procreation (Waldby, 2015). Cryopreservation is a key technology for this practice.

Cryopreservation is the use of very low temperatures (most commonly − 196 °C) to preserve and store structurally intact living cells and tissues (Gosden, 2014). At such low temperatures, all biological activity stops, including the biochemical reactions that lead to cell death and DNA degradation (ibid). Cryobiology has become a central technique in contemporary life-sciences as more and more types of tissue and cellular material can be frozen and thawed with almost no loss of vitality (Waldby, 2015). This is particularly relevant when it comes to assisted fertility practices as it allows the preservation and further use of different materials (sperm, eggs, gonadal tissues, and embryos) for reproductive purposes (Lemke, 2019). We refer to these applications as cryo-fertility. Cryo-fertility has become a key technology of fertility preservation for patients (both male and female) facing the risk of sterilization/infertility, for example due cancer treatments, but now also for ‘social’ reasons.

Pertinent academic debates on cryo-fertility and temporality highlight: the way such technologies halt the temporality of reproductive biology and the related social and ethical implications (Kroløkke et al., 2020; Landecker, 2010; Lemke, 2019, 2021); the use of such technologies in order to control fertility while synchronizing different temporalities (e.g. social and biological) (Baldwin, 2019; Brown & Patrick, 2018; Kroløkke, 2019; Waldby, 2015); and the analysis of the criticism directed at such technologies as reflecting moral and normative perceptions concerning the “appropriate” timetables and life-course ideals (Rimon-Zarfaty & Schweda, 2019). What are still missing in this debate are the socio-cultural aspects directing such reproductive temporalities. The impetus behind this research was therefore to embrace a cross-cultural perspective for gaining an understanding regarding if and how different socio-cultural contexts create and reflect different time constructions in the context of cryo-fertility.

In this paper we aim to expand our understanding of the way temporality emerges and is negotiated in the contemporary practice of cryopreservation by focusing on cryo-fertility and especially social egg freezing (‘SEF’). Starting with a brief historical overview of the main stages of cryo-fertility, we indicate the intersection or co-production of technology and societal norms. In what follows, we theoretically reflect on cryo-technologies as creating new epistemic perspectives interconnecting biology and temporality, while drawing insights regarding temporality in contemporary egg freezing practice. In the next section, we present empirical findings from a qualitative study using interviews of the case of SEF users in Germany and Israel to illustrate different reproductive temporalities, the way these are negotiated in a specific application of cryo-fertility, and reflected in different socio-cultural contexts. The analysis of the interviews highlights three emerging formations of temporality that inform attitudes towards SEF: the ‘extended present’, ‘waiting’, and ‘reproductive futurism’. In the final concluding section, we focus on the theoretical concept of ‘cryopolitics’ and explore the theoretical implications of a gendered and culturally sensitive reconstruction of medical technological innovations and related reproductive temporalities and how they can inform philosophical and bioethical debates on cryobiology.

## Historical stages of cryo-fertility as example of co-production of technological and societal interest

While the impact and implications of different biotechnological innovations such as cloning, organ transplantation, tissue generation, and artificial insemination attracted much scholarly attention and academic debate, the history and influence of cryobiology and cryopreservation technologies (which are fundamental to each of those biotechnological innovations) within the life-sciences have been, to a large extent neglected (Lemke, 2019; Radin, 2013). These currently attract various scholars due to the particular epistemic and social implications of ‘freezing as suspension’ (see below). We will summarize here some basic stages of technology development to indicate how such development can be understood based on co-production from scientific and societal efforts.

Interestingly, some of the most important developments and breakthrough in cryopreservation technologies took place in the context of reproductive medicine and related scientific experiments (Gosden, 2014), particularly in the fields of animal agriculture and breeding (Kroløkke, 2019; Waldby, 2015).

Up to the early 1900s, all attempts to preserve cells at subzero temperatures had failed and were therefore deemed almost impossible (Gook, 2011). Starting with the collection of sperm for artificial insemination by a donor in the first half of the twentieth century, the question of ‘preservation’ became more and more relevant (Daniels & Golden, 2004). In 1937, the biologist B.L. Luyet formulated a biological scientific protocol elaborating the principles of cell cryopreservation. While his experiments were only partially successful, he advanced the field from being merely speculative into a proper scientific foundation, thus marking an important milestone (Gosden, 2014). Luyet was the first scientist to experiment on the usage of ultra-rapid cooling rates, already in 1937, and in 1938, to identify the beneficial effect of dehydration prior to freezing (Gook, 2011).

Another important milestone in the development of vitrification techniques was made in the late 1940s by a research team consisting of Sir Alan Parkes (a reproductive biologist), the cryobiologist Audrey Smith, and their graduate student Christopher Polge, who later became a prominent researcher in the field. Interestingly, the team focused on laboratory experiments in sperm cryopreservation (chicken, rabbit, and human spermatozoa). They chose to focus on spermatozoa both because their movement served as a natural viability indicator and due to the practical value for the cattle breeding industry. The experiments resulted in the accidental discovery of the cryoprotective properties of glycerol (Gosden, 2014; Polge et al., 1949)- constituting an important turning point in cryobiology in general and cryofertility, in particular (Gook, 2011; Gosden, 2014).

During the 1950s, this important discovery and the possibility to freeze human sperm led to the establishment of sperm banks, mainly for men wishing to preserve their sperm due to cancer treatments or before undergoing vasectomy (Gosden, 2014). In 1954, the first birth following the use of human cryopreserved spermatozoa was reported (Gook, 2011). Later, a whole US industry of sperm banking and, triggered by positive eugenics, a sperm bank of “geniuses”, shaped the new picture of “high-quality sperm”, always available (Daniels & Golden, 2004).

Another important turning point in the development and application of cryo-fertility was marked by in-vitro-fertilization (IVF). When IVF was first introduced in the late 1970s, the cryopreservation of in-vitro embryos or eggs was still unavailable. The early treatments therefore followed the natural female menstrual cycles in which (usually) only one oocyte was available for insemination. Very soon, fertility experts raised the idea of improving the success rates of the treatment by increasing the number of available oocytes, i.e., by using hormonal treatment generating ovarian stimulation. This procedure, which later has become a standard practice, led to the creation of a multiple number of embryos per treatment cycle (Gosden, 2014). The first pregnancy from a frozen human embryo was reported in 1983, and the first live birth from IVF using a cryopreserved embryo was reported in 1984 (Wang & Sauer, 2006). However, the creation of superfluous embryos created also “an acute ethical dilemma” (ibid, p. 262). Debates started whether to transfer only one or a few embryos and to discard the rest. This raised the issue of the moral status of the embryonic entity (Gosden, 2014; Wang & Sauer, 2006). Hence, Western countries developed a wide range of different legislation regulating the creation (e.g., in terms of how many embryos can be produced in vitro) and the acceptable usage of such entities, as well as strategies for overcoming the problem of embryo destruction (Hashiloni-Dolev, 2013) by freezing the fertilized oocyte in a pre-embryo stage. The possibility of freezing embryos has in turn led to ethical and legal debates, concerning whether the manipulation of the beginning of human life is morally acceptable (Hashiloni-Dolev & Schicktanz, 2017), whether there are potential health risks for the future child (Michelmann & Nayudu, 2006) and how cryopreservation might limit the success rates (Gosden, 2014; Michelmann & Nayudu, 2006; Wang & Sauer, 2006). Further issues (which became more apparent also with the rise of embryonic stem cell research), included custody, ownership, and responsibility (ESHRE, 2001).

Another milestone in the historical evolution of cryo-fertility practices was the technological development of ‘vitrification’ or so called ‘fast freezing’. The earlier ‘slow freezing’ is a gradual cooling technique which slowly cools the cell/tissue but leads to ice crystals which can harm the tissue/cells’ potential after thawing. By contrast, vitrification technique combines ultra-rapid cooling with use of high concentrations of cryoprotectants^1^ allowing a rapid entry into a glass-like state while avoiding crystallization. Vitrification therefore increases the chances that the cell/tissue will be viable and functional when thawed. This is especially important for egg freezing since eggs are large cells with a high content of water (Gook, 2011; Gosden, 2014).

While embryo freezing quite quickly became a standard practice in IVF clinics, oocytes are more difficult to cryopreserve (Gosden, 2014). During the mid-1980s, there were few reports of viable pregnancies using frozen oocytes^2^ but then the practice almost disappeared for a decade (ibid), mainly due to inconsistent results and concerns regarding embryonic development and the health of the future children (Gook, 2011). During the 1990s, egg freezing continued to be of interest to several researchers who focused on improving freezing methodologies and post-thaw survival rates as well as the normality of embryo development from a previously frozen egg (ibid).^3^

This type of research and development can also be identified as influenced by politics and ethics. For example, substantial research in this field was promoted by Italian researchers in the context of Italy's Law 40- approved by Parliament in 2004. The law restricted PGD (pre-implantation genetic diagnosis) along with embryo freezing, embryo research, and egg donation (Baldwin et al., 2014; Gook, 2011; Martin, 2010). The restriction of embryo freezing has been identified as encouraging further technical advances in oocytes cryopreservation (Martin, 2010). In other places, egg freezing was perceived as sidestepping controversies around embryo disposition, including custody issues, “orphan” embryos, and issues related to embryonic research and the exploitive dynamics involved in egg donation (ibid).

Towards the end of the 1990s, egg freezing appeared as a new option for fertility preservation but was mainly used for medical reasons—i.e., for women who will potentially sustain partial or total loss of fertility (e.g., due to cancer treatments) (Wang & Sauer, 2006). In 1994, the world’s first egg bank was established in Melbourne, Australia, at the Royal Women’s Hospital to preserve fertility for women with malignant diseases (Gook, 2011).

The most recent milestone in the context of egg freezing was reached in 2012, when both the American Society for Reproductive Medicine (ASRM), and the European Society of Human Reproduction and Embryology (ESHRE) decided to lift the experimental label of the procedure. The professional-ethical move paved the way for using the procedure for preserving fertility due to so called ‘social’ reasons—what has been recognized as ‘non-medical’ or ‘social’ egg freezing (ESHRE, 2012; ASRM & SART, 2013; ASRM, 2018). Egg freezing is therefore increasingly used to enable healthy women to prolong their fertility. In the US for example, reports from the Society for Assisted Reproductive Technology indicate that in 2013 (the year following the ASRM approval), reporting clinics performed approximately 5000 cycles of egg freezing. Those numbers were already doubled in 2018, when approximately 11,000 cycles were reported (Birenbaum-Carmeli et al., 2021).

## Between cryo, biology and temporality

Cryopreservation technologies rely on the ability to preserve biological materials in extreme sub-zero temperature. They therefore encompass a unique configuration of temperature and temporality (Oikkonen, 2020). Indeed, several scholars share the interpretation of cryo-technological developments as introducing new perceptions of time, biology and their interrelation, calling for empirical investigation (Kroløkke, 2019, 2021; Kroløkke et al., 2020; Lemke, 2019).

In her research on cryopathy and cryonics, Kroløkke (2021, pp. 35–36) offers a multimodal domain entangling different types of cold: that is ‘natural’—(ice) and ‘artificial’—(cryo). Following this conceptualization, in the context of cryo-fertility, and especially egg freezing, ‘natural’ ice crystal formations have been positioned as a threat (that should be avoided by the usage of vitrification and cryoprotectants). In this sense the technoscientific possibility to replace the ‘natural’ with ‘artificial ‘ cold (cryo), becomes, or is understood as holding value, as it (allegedly) promises to challenge biological temporality.

Reflecting upon the common metaphor of the *‘biological*-*clock*’ which encompasses ideas of ‘natural’ temporal limitations, the development of cryobiology (i.e. the ability to generate artificial cold) can be perceived as challenging biological temporality, as reshaping the boundaries between life and death, health and illness, youthfulness and aging, mortality and generativity (Katz et al., 2020; Kroløkke, 2021), and thus as inspiring or introducing new epistemological perspectives. As Hannah Landecker (2010) observes, cryobiology changes what it means to be biological. Formerly, being ‘biological’ meant to be embedded in the circle of continuous life process including being born and dying. Now being biological, cellular, or alive also allows “to be suspendable, interruptible and storable, freezable in parts” (ibid, p. 217). In other words, the use of cryobiology—the ability to stop and start biology with its bounded temporality or clocks, to arrest and suspend cellular activity and reanimate it at some future date, calls for a different perspective on the relationship between biology and time and the ability to synchronize and facilitate temporality (Kroløkke, 2019; Waldby, 2015). When it comes to temporality, the “plasticity of living matter” (Landecker, 2010, p. 219), and the manipulation of the plastic matter of the organism while halting it at a certain state, results in “things living differently in time” (ibid). Therefore, freezing technologies have been referred to as allowing ‘time travel’ (Katz et al., 2020), as a form of biological ‘time machine’ (Kroløkke, 2019) or ‘temporal prothesis’ (Radin & Kowal, 2017), and thus as enabling new ontologies- otherwise unavailable (Oikkonen, 2020).

Within this context, cryobiological technologies have further been identified as creating a new form of decontextualized ‘latent life’ (Radin, 2013) or ‘suspended life’. By enabling vital processes to be kept in a liminal state cryo-technologies create a new condition in which biological entities are neither alive nor dead (Lemke, 2019)—“an inert life without (apparent) change” (Lemke, 2021, p. 11). The terminology of ‘suspense’ and ‘suspension’ was also offered by medical anthropologist Klaus Hoeyer (2017). According to Hoeyer, the term ‘suspense’ holds several meanings in this context: putting biological parts on hold, their suspension from the body from which they were taken, and the resulting suspension of life and death (ibid).

The state of latency or suspense in itself also holds an integral temporal orientation as it seeks to ensure the past, in the sense of preserving an entity’s state at a given point in time long after this point has passed, while being oriented towards the future by keeping such biological materials available for future use (Kroløkke, 2019). Reflecting on the concepts of time and reversibility, Lemke (2021), further relates to ‘suspended life’ as introducing a new time configuration which extends the present towards the future. According to him, the expansion of the duration of the present might in turn “delay changes or postpone necessary decisions” (ibid, p. 9) by offering reversibility. At the same time, cryobiology can also be analyzed as representing a scientific effort to manage the future (Radin, 2013), reflecting a more comprehensive ’regime of anticipation‘ (Adams et al., 2009). This regime which currently guides various technoscientific and biomedical practices involves a temporal epistemological dimension perceiving the future as open and incidental while at the same time depending on present actions (Lemke, 2019, p. 453). Within this ’regime of anticipation,’ the “sciences of the actual” are replaced by a predictive or even “speculative forecast” (Adams et al., 2009, p. 247). These modes of anticipation are closely linked to emotions and rationalities of responsibility, prevention, and preparedness: They involve a complex set of concerns, fears, and hope “linking epistemic orientations to moral imperatives” (Lemke, 2019, p. 453). A similar idea was highlighted by Hoeyer (2017) who claimed that the suspension of biological decay via cryopreservation results in the creation of “a space for action in which new social forms are built, new property managements emerge, and new hopes and concerns can flourish” (p. 209) (for relevant discussion see: Kroløkke et al., 2020). Thus, the frozen material is constructed as a form of ‘promissory capital’ (Lemke, 2019; Thompsom, 2005), and the ability to freeze biological material draws on the promise or hope of future revival. It therefore also becomes a type of insurance policy—a protection of life against death (Waldby, 2015).

Finally, the usage of ‘artificial’ cold (cryo) for reshaping or challenging life, its boundaries and temporality can be further conceptualized using the theoretical framework of ‘cryopolitics’. When studying the political and social impact of the life sciences and biomedical practices and the way life is extendedly regulated by technoscientific means, social scientists often draw on the Foucauldian concept of ‘biopolitics’ (Foucault, 1978, 2003; for relevant discussion see: Lemke, 2019). Within this general theoretical framework, and due to the rapid developments in cryo-technologies, the term ‘cryopolitics’ (Radin & Kowal, 2017) has been suggested to conceptualize the politics of low temperatures, suggesting that cryo-technologies have become a main biopolitical tool of the twenty-first century (ibid; Kroløkke, 2019). Cryopolitics refers to the socio-political aspects and related mechanisms or “strategies of generating, regulating and processing ‘suspended life’” (Lemke, 2019, p. 454). While drawing on the concept of biopolitics, Radin and Kowal (2017, p. 6) emphasize the important intensification and even intervention represented by the concept ‘cryopolitics’. According to them, while the Foucauldian concept of biopolitics pertains to the way power “makes live and let die”, the concept of cryopolitics, which draws on a more recent scientific discourse and practice while suspending animation and action, produces a zone of existence emphasizing the centrality of “make live and not let die” (for relevant discussion see: Kroløkke, 2019; Lemke, 2019, 2021; Kroløkke et al., 2020). In this sense, cryopreserved materials (that is organisms or bits of their bodies) are exposed to “a new onto-political regime, being neither fully alive nor dead” (Lemke, 2019, p. 455). According to Radin and Kowal (2017, p. 12), cryopolitics therefore focuses on the usage of ‘artificial’ cold (cryo) to reorient life- as well as related perceptions concerning what life is- in time.

## Temporality in contemporary practice of cryopreservation: The case of ‘social egg freezing’ (SEF)

When it comes to social egg freezing, cryopreservation sees to facilitate reproductive plasticity (Kroløkke, 2019). It aims to enable women to disconnect their reproductive potential from its biological rhythms in the hope to secure their reproductive future, i.e., by providing ‘young’ eggs for later life. Egg freezing thus represents a specific example of the ‘regime of anticipation’ referring to the frozen oocytes as a unique ‘promissory capital’ in the form of post-menopausal reproductive potential, reconstructing reproductive temporal horizons. The procedure may therefore be analyzed as a technological attempt to stop women's ‘biological clock’, ‘freeze time’ or else synchronize different temporal orders.

As described by Kroløkke (2019), the case of oocytes freezing entangles “the somatic bodily temporalities (growing old/becoming infertile) with institutional temporalities (how society structures procreation in women’s lives), normative temporalities (when a woman is viewed as too young or too old to procreate) and affective temporalities (hoping to become a parent or fearing it is too late)” (Kroløkke, 2019, p. 530). Indeed, previous empirical research focusing on SEF users demonstrated how freezing seemingly becomes a form of biological ‘time prosthesis’ (ibid; Kroløkke et al., 2020), aimed at reconciling ‘social’ and ‘biological’ temporalities (Waldby, 2015).

Reproductive decisions regarding time, timing and life planning are therefore not only embedded in somatic and biological ‘clocks’, but also reflect multiple perceptions of time that are normative and socially constructed (Nowotny, 1992). In other words, individual life is not just a biological process that follows a certain biological clock, but a sequence of phases and thresholds reflecting socially constructed life course and biographical ideals, as well as generational roles and statuses reflecting collective schedules (Rimon-Zarfaty & Schweda, 2019).

Within this context, SEF can be perceived on the one hand as a type of reproductive management reflecting normative ideals such as empowerment by control over the temporality of reproduction (Robertson, 2014). On the other hand, it also involves normative uncertainty about what is the ‘right’ timing? Public and scholarly critique evoked by SEF demonstrate the existence of particular social expectations towards women, motherhood, and the “ideal” life course. This frames SEF as a deviance from collective temporal reproductive constructions according to which pregnancy is supposed to take place at a certain age or during a particular stage of the life course (Baldwin et al., 2014; Bozzaro, 2018; Bühler, 2015; Weber-Guskar, 2018). SEF has therefore triggered a controversy around “late” or “old” mothers (see for counterarguments: Bernstein & Wiesemann, 2014; Smajdor, 2009). Linked to this, concerns have been raised regarding the alleged harm to and burden on children of “old mothers”. Further, concerns include the latter’s ability to fulfil customary parental roles and responsibilities, the burdens created by early filial care responsibilities, etc. (see for relevant discussion: Rimon-Zarfaty & Schweda, 2019; Kroløkke et al., 2020).

However, little is known about if and how temporality is concretely constructed in the case of SEF. Therefore, a leading research question for us is how temporality emerges and takes shape in its context. Our case-study elaborates theoretical links between socio-cultural and normative meanings of time while examining the implications for those affected- that is the women using SEF. While during the interviews, our interviewees did not relate to the cryo-technology and its properties as such nor to the related theoretical conceptualization of human biology and temporality (apparent in the scholarly debates), they nevertheless highlight their everyday expressions and representations. We therefore focus on those ‘lay moralities’(Raz & Schicktanz, 2009a, b) and their yet neglected cultural interpretations. By analyzing the views and experiences of SEF users in Germany and Israel, we attempt to gain insights into the different reproductive temporalities, how these are morally guided by ideals, hopes or concerns, and the ways those may differentiate between different societies.

## Reproductive temporalities and SEF in Germany and Israel

In the following, we present results from an empirical study analyzing 39 qualitative personal in-depth semi-structured interviews (conducted during 2018–19): 23 interviews were conducted with Israeli SEF users and 16 with German SEF users.^4^

The interviews lasted 3–1.5 h and were conducted in a location preferred by the interviewees. Interviews were conducted in Hebrew in Israel and in German or in English in Germany (as preferred by the interviewee). A similar interview guide was used in both countries. Interviews in Hebrew and English were conducted by Nitzan Rimon-Zarfaty. Interviews in German were conducted by a research assistant (Ms. Lisa-Katharina Sismuth). In case it was preferred and requested by the interviewee, the interview was conducted via the telephone or online platform. Interviewees were asked about their motivations to use egg freezing (including timing issues and family planning), their main considerations and the perceived advantages and burdens of SEF. They were also asked how they experienced the interaction with the medical staff and the consultation they received prior and during the procedure. They were further asked about the experience of the use of SEF, the reactions of their social environment, their opinion regarding the current regulation of SEF in their country and the overall public debate.

Interviewees were recruited using recruitment flyers placed in fertility clinics (upon the clinics’ consent), via relevant internet forums (following the consent of the forums’ managers) and using a snowball sampling method.

Interviews were tape recorded (with the participants’ permission) and fully transcribed. Quotes were later translated into English. Interviews conducted in German were fully translated into English.^5^ Transcripts were analyzed to uncover discursive themes and categories of themes recurring within and across national groups (Denzin & Lincoln, 1994). The coding and thematization process was generally based on the constructivist version (Charmaz, 2002) of the grounded theory approach to data analysis (Glaser & Strauss, 1967; Strauss & Corbin, 1990). Emergent topics identified through inductive coding were added to the analysis and compared between the different national-cultural groups. This enabled us to detect relevant considerations, ideas and moral arguments that can be further interpreted in relation to reproductive temporalities and the related cultural scripts. As an international (German-Israeli) and inter-disciplinary research team, and drawing on collaboration across research nationals and cultures, our interpretations and analysis were discussed and peer reviewed for reflexive insights.

Our cross-cultural comparative research framework was aimed at deconstructing the often implicit, taken-for-granted assumptions concerning time, timing, and planning, and unveil their underlying cultural narratives. Previous comparative research on bioethical issues between Israel and Germany identified both countries as representing two opposing regulatory frameworks and professional cultures in biomedicine in the context of the beginning and the end of life (e.g., Hashiloni-Dolev, 2007; Hashiloni-Dolev & Shkedi, 2007; Raz & Schicktanz, 2009b, 2016). Both contexts are of high relevance for human temporality, namely life-course, planning, and ‘timing’. In relation to reproductive technologies, the German regulatory framework (originated at the Embryo Protection Act (EPA)) was identified as rather restrictive, while the Israeli regulation has been identified as permissive (Hashiloni-Dolev, 2007; Hashiloni-Dolev & Shkedi, 2007). Moreover, Israel is one of the first countries that officially regulated SEF. The regulation, issued in September 2010, allows freezing the eggs of healthy women aged 30–41 years and the implementation of fertilized eggs until the age of 54. The procedure is limited to 20 frozen eggs or 4 cycles. The procedure needs to be paid out of one’s own pocket. In Germany, by contrast, there is no formal legal or regulatory framework of SEF—which is allowed and performed (Rimon-Zarfaty et al., 2021). The German EPA, which prohibits egg donation, permits the freezing of fertilized eggs only at the pronuclear state (while limiting the cryopreservation of embryos), and therefore does not restrict the freezing of unfertilized eggs (Robertson, 2014).

Overall, our cross-cultural comparison, which allowed for reflecting on the similarities and differences across societies, reveals three main types of anticipatory motivations for using SEF: postponing reproductive decisions, “waiting” for a partner, and usage of SEF in the hope for or planning of a multiple number of children. Those three motivations are not exhaustive nor mutually exclusive –and thus can possibly characterize a single interviewee.^6^ We will present and discuss each of those motivations and offer a theoretical conceptualization of the emerging temporalities, including the ‘extended present’, ‘waiting’, and ‘reproductive futurism’ respectively.

### Postponing reproductive decisions: extending the ‘extended present’

One of the cross-cultural differences apparent among our interviewees revolved around the idea of motherhood and the decision to become a mother. While most of the Israeli interviewees expressed a wish to have children, when it came to the German interviewees, the decision to become a mother seemed less obvious and more conflictual. Several issues and concerns were raised in the interviews as related to the difficulty in making this life-changing decision. The first concern brought up by German interviewees revolved around ideas of readiness. They raised ideas of “right time” for becoming a mother which they connected to certain milestones or life conditions (Baldwin et al., 2015) they believed should be reached: a stable relationship, financial stability, and emotional readiness—all of which may also reflect broader social expectations. The second concern revolved around the German labor market which is perceived by many of the interviewees as intense and burdensome and as therefore discouraging motherhood. The educational and training courses were portrayed as long and strict. Although not serving as a direct reason for performing SEF- that is, interviewees did not choose to use SEF due to career considerations, they still acknowledged this issue and its effect on their reproductive decision making. It therefore seems that those women try to cope with two highly gendered time conflicts: a work-family conflict (Daly & Bewley, 2013; Waldby & Cooper, 2008) and a related biological-social time conflict (Leccardi, 2005a).

A third major concern highlighted how relationship-formations are nowadays challenged. Interviewees emphasized the difficulty to form a stable relationship in today’s dating world with its instability and endless possibilities. Finally, German SEF users also mentioned a strong social expectation towards German mothers (mainly in Western-Germany), expecting them to put all their energies into motherhood, and a related social de-legitimation and stigmatization of working mothers. The concept of ‘Rabenmutter’ (Raven Mother) was frequently mentioned as representing such “neglectful” mothers. Some of our interviewees mentioned that this message is also supported by existing regulation which supports mothers in staying at home (e.g., long maternity leaves, tax legislation and the shortage of childcare provision) (Fagnani, 2002; Mckay, 2011; Bauernschuster & Rainer, 2012).

The concerns raised by our interviewees can be further discussed within the framework of changes faced by women today. Those include a complex combination of changes in career and educational paths (Leccardi, 2005b) which comprise of new labor-market requirements regarding higher flexibility, longer training periods, and less occupational stability (Bozzaro, 2018; Waldby & Cooper, 2008); changes in relationship patterns and formations leading to difficulties in forming commitment and stable relationships (Bozzaro, 2018; Illouz, 1997, 2012; Inhorn, 2020); as well as high social expectations from German mothers (Fagnani, 2002; Mckay, 2011). All these issues serve as a background for understanding reproductive postponing and a notion of a temporal gap between the biologically bound time of women’s reproduction and a newly evolved social time (Waldby, 2015).

Against this background, one main motivation for using SEF emerging from the interviews—also reflecting a form of reproductive temporality—is to postpone reproduction and even more generally, reproductive decision making. This motivation characterized a minority of the interviewees, mainly Germans. By contrast, the decision to have a child came up as almost unconditional from most of the Israeli interviewees (with only very few exceptions). Israeli interviewees also acknowledged the current iterative structures of relationship formation and career patterns, yet those did not seem to raise concerns to a similar extent regarding the related time conflicts and the basic decision to become a mother. As it was put by two Israeli interviewees a 33 and a 34 years old respectively:

“In Israel the dogmatic decision is (…) to become a mother. (…) the default is to be a mother”

“From a very young age I wanted a big family, it is something I grew up on (…) very natural”

In contrast, it seems that for our German interviewees the question of whether to have children is more open and at times more conflictual. Therefore, the option of postponing the decision to become a mother as such, became relevant. This point was exemplified by one of our German interviewees, a 37-year-old, when talking about her main motivations for using SEF and the main advantage of the procedure as she perceives it:

“Time was flying and I’m, (…) 38 (…) there is not that much time left to take an active decision (…). I cannot take the decision now if I wanna have a family (…). I think the advantage [of SEF] is (…) that you as a woman have more flexibility to integrate or schedule (…) this family topic into your life. You just prolong the phase that it (…) might be possible. I’m a kind of busy person. I love my job; I love all the things I do. (…) might sound very egoistic. It’s basically the decision of changing my lifestyle”.

As exemplified by this quotation, this interviewee is not sure whether she is willing to make the life-changing decision of becoming a mother. While other interviewees expressed more ambivalence or difficulty with their inability to reach such a decision, she explains that she enjoys the current status quo of her life, her job, flexibility, hobbies, and aspirations. While acknowledging the limitations of SEF, she therefore uses it to gain more time until she will be forced to face such decisions.

Following this line of reasoning, the motivation to maintain the current status quo and gain more time for making reproductive decisions can be generally conceptualized within the idea of an ‘extended present’ (Leccardi, 2005a; Nowotny, 1985). Expecting a potential contradictory nature of motherhood with their public sphere/social life, self-realization or personal tendencies, these women anticipate a future biographic discontinuity due to motherhood. As a result, they abandon the medium-long term future and concentrate on the time dimension of the ‘extended present’ in which they are able to make short- or medium-term plans. SEF therefore serves as medical-technological mean for further extending the extended present by postponing existential questions and definitive decisions, while preserving future choices (Rimon-Zarfaty & Schweda, 2019; For similar observations in the context of Belgian social egg freezing users and British medical egg freezing users see: De Proost & Paton, 2022). Our finding further highlights the connection between the medico-technological cessation of vital metabolic activities and the idea of continuous present in turn enabling the postponement of personal decisions concerning “the concrete “when” of the “whenever”” (Lemke, 2021, p. 12).

Furthermore, as this quotation demonstrates, German women using SEF are at least to a certain extent also faced with social criticism identifying them as “egoistic” or “selfish” as they might be perceived as prioritizing career and self-realization at the expense of having children. Such perceptions can also be connected to ideals of “proper” reproductive timing. Postponing reproductive decisions can thus be identified as representing a “prolonged” transition to adulthood. In a similar manner, such criticism is also connected to a controversy around ‘late motherhood’.^7^ This type of criticism serves as an indication for implicit normatively loaded and gendered ideas of the ideal female life-course (Weber-Guskar, 2018; Rimon-Zarfaty & Schweda, 2019). By extending the ‘extended present’, SEF therefore challenges traditional age norms, phase ideals and biographical schedules. Indeed, while attempting to extend the extended present, many of our interviewees have been struggling with the idea that they are “off time” or “out of sync” (Baldwin, 2019). Such concerns will be further discussed in the context of our second type of interviewees highlighting the use of SEF in the context of ‘waiting’.

### ‘Waiting’ (?) for a partner

Unlike media representations which often portray SEF users as deliberately delaying childbirth in order to achieve educational or career goals (Baldwin, 2017; Martin, 2010), current empirical research, e.g., from Israel and the US (Inhorn et al., 2018), Germany (von Wolff et al., 2015), the US (Brown & Patrick, 2018; Carroll & Kroløkke, 2017), the Netherlands (de Groot et al., 2016), Turkey (Kılıç & Göçmen, 2018), Belgium (De Proost & Paton, 2022) and the UK (Baldwin et. al., 2015; Baldwin, 2019), suggests that most women who opt for SEF are middle-class highly educated single women who make use of the technology mainly due to lack of partner (for relevant analysis see: Inhorn et al., 2018; Inhorn, 2020).

Indeed, the majority of our interviewees from both Germany and Israel shared the wish to have children in a relationship, i.e., with a suitable partner, as a main motivation for using SEF. Both German and Israeli SEF users sharing this motivation explained that at this point, they do not have a partner – a situation in which they did not want to remain. Facing the ticking ‘biological clock’, they feel they are “running out of time”, or—as it was put by one of the German interviewees—are at a point of “end time panic “ (see also: Baldwin, 2019). Generally, the status of (late) singlehood or lack of a partner came up extremely prominently, at times accompanied by expressions of loneliness and anxiety. They hope that SEF will buy them the extra time to enable them to find a partner, have a child within the framework of a relationship, and get back on the life course track. This motivation is exemplified by one of our Israeli interviewees, a 39 years old woman:

“It will give me an option (…) I will (…) find the partner that will be a good match for me. (…) and this dead-end of fertility, somehow becomes a bit softer. (…) it does not turn the situation into something else but it helps, especially in the process of looking for a relationship (…) a bit of air”.

As this quotation demonstrates, this interviewee chose to use SEF to “get some air”—that is a break or a ‘time-out’ from the ticking ‘biological clock’ that will enable her to find a partner and form a relationship within which she can fulfill her wish to have children. A similar motivation was brought up by a 29 years old German interviewee:

“When you’re like 34/35 and you don’t have a partner (…) you really are in kind of a rush, you know? Like: Ok, Hey, my name is … so let’s have children. (…) This gives you also a bit of a laid—back attitude when it comes to relationships”.

One cross-cultural difference detected within this identified type of SEF users, has to do with the option of embryo-freezing. While embryo freezing is restricted in Germany, the Israeli regulatory framework enables women opting for SEF also to freeze embryos (fertilized eggs), using donor sperm. As came up in our interviews, the option of embryo freezing, is promoted by certain fertility experts who claim more experience and higher success rates than with egg freezing. This option confronted our interviewees with the question of whether or not to “commit” to a donor sperm while also bringing up the possibility of single motherhood. Importantly, while longing for a partner, some of the Israeli (mainly secular) interviewees chose to freeze both embryos and eggs, in the hope of securing their chances for future motherhood- also relating to this choice as a last resort or a ‘plan b to the plan b’. These findings correspond with the Israeli wish for motherhood discussed earlier.

The case study of SEF therefore, generally brings up the status of singlehood as a main category—in ways which highlight the temporal collective organization of social life—meaning the fact that there are certain socially accepted time-frames or age norms within which women are expected to engage in a stable relationship (Lahad, 2012) and have children. While our findings show how social understandings regarding the “appropriate” timing and duration of this life phase can vary (as demonstrated by the reported German controversy around ‘late motherhood’), singlehood nevertheless represents an overarching liminal, temporary, and transitory stage (ibid).

In the context of singlehood, SEF users’ experiences and understandings can be interpreted within the framework of *waiting* (For similar observations in the context of American SEF users see: Inhorn, 2020). Smith-Hefner and Inhorn (2020) refer in this context to a “state of waithood” (p. 3), in which one is waiting (in the sense of unintentional/unexpected delay) to marry and have children. The context of SEF usage is however, ambivalent and two-fold. When relating to the temporal constituents of the female self, Pickard (2020), who presents an overview of relevant feminist scholarship, discusses its identification as reflecting a gendered tension between constraining modes of ‘waiting’ or ‘expectation’ versus aspects of ‘choice’ and agency- to which we can relate as empowering. On the one hand, women are subjected to the highly gendered notion of “patiently waiting”, reflecting a traditional and constrained temporal condition. This temporal notion becomes particularly salient in the context of traditional heterosexual romantic scripts according to which single women are expected to wait to be chosen (Pickard, 2020), or more generally wait for a partner. Following this line of reasoning, according to Lahad (2017), the notion of ‘waiting’ attributed to single women, reflects a heteronormative logic which produces power relations supported by a disciplinary temporal regime. At the same time, and in line with Pickard’s (2020) insights regarding female temporality in late modernity, as our findings reveal, while women’s temporal experiences can be interpreted as reflecting a traditional style of ’waiting‘, those experiences are also simultaneously counterposed by the neoliberal emphasis on ‘choice’ and agency-reflected in women’s search for a right partner and demanding a form of control over their reproductive potential. This dialectic “results in a chronic state of ambivalence” (ibid, p. 314). On the one hand, as single women, our interviewees are subjected to the traditional idea that they should wait for the right partner. Such gendered temporalities and the related constitution of the hybrid feminine self, underlie the stalling and slowing down of the “gender revolution” (Pickard, 2020). On the other hand, within the Western capitalist social context idealizing notions of efficiency, such waiting is understood as a waste of time that should be eliminated (Lahad, 2012). Following this line of reasoning, the threat is that single women will overly extend their waiting time and “miss the train” with no possibility to rejoin the collective temporal linear path (ibid). Our interviewees therefore attempt to avoid additional ‘waste of time’—they use SEF in the hope that it will give them the ability to synchronize and facilitate temporality and thus claim a form of reproductive agency (Brown & Patrick, 2018; Lahad, 2017)- identified as empowering- which in turn may also enable them to “look for”, “find” or “choose” the right partner.

SEF users are therefore faced with a dialectic tension between choice and agency and the traditional expectations of waiting- which also represent competing interpretations in the context of SEF usage. This tension echoes the gendered challenge of the dual management of a rational-instrumental approach to time, associated with the future, and the caring time of immanence (Pickard, 2020, p. 318). Within this context, SEF serves as an empowering means by which women are able to mobilize greater agency over their reproduction—also reflecting a positive moral attitude towards active and “responsible” (hopefully) efficient time management in the light of future prognoses and risks (i.e., of ‘biological’ fertility decline) (van Carroll & Kroløkke, 2017; de Wiel, 2015). In this sense, instead of passively accepting the inevitable decline of their fertility, SEF users take action/responsibility/control to preserve their reproductive future options (Baldwin, 2019). Such an attempt can also be linked to their wish to avoid future regret and blame for not taking action (ibid; Baldwin et al., 2019). However, the use of SEF paradoxically produces a form of temporal stalling, enabling SEF users to extend their waiting time (see also Bozzaro, this issue). In this sense, the use of reproductive technologies (in this context, SEF), reflects the interplay between intentionality and constraint as it on the one hand a manifestation of intentional, future oriented agency, while at the same time a mean for prolonging a gendered state of ‘waiting’ and ‘expectation’ (Pickard, 2020).

Acknowledging this paradox and the way it is reflected in SEF users’ experiences and motivation, it therefore seems that the multiple experiences of women’s lives cannot be reduced to dichotomous binary categories (Lahad, 2017, p. 16) but rather represent a complex and inconsistent negotiation between empowerment and constraint. From this perspective, SEF, which challenges traditional age norms (Rimon-Zarfaty & Schweda, 2019), and even to a certain extent family models (as exemplified by the Israeli interpretation of SEF as including embryo freezing, thus creating a negotiation around single motherhood), holds an empowering potential and experience. Nevertheless, at the same time, the context of singlehood emphasizes the notion of ‘waiting’. When women use SEF to buy more time for finding a partner—namely a man to have children with, egg freezing becomes a technological concession to unintentional ‘reproductive waithood’ (Inhorn, 2020). In this sense, women use this novel technology to meet traditional cultural familial scripts with their biographical scenarios and related timelines (Inhorn, 2020) (see also Bozzaro, this issue).

### Long term planning- ‘reproductive futurism’

The third motivation for using SEF emerging from the interviews is common among a distinct minority group of interviewees, all of them Israeli Jewish-religious women (who self-identified as observant to their faith). Unlike the (mainly Catholic) Christian idea of the ‘divine order of creation’ (Schöpfungsordnung), naturalness and refrain from ‘playing god’ as an intervention in the sacred act of creation (Bühler, 2015; Hashiloni-Dolev, 2013), the Jewish tradition perceives SEF as unproblematic. In fact, in Israel, SEF is supported by certain rabbinic authorities as an alternative to single motherhood^8^ (Inhorn et al., 2020). Therefore, SEF becomes attractive for Jewish-religious single women wishing to have children within a traditional heteronormative family model.

These women usually start to use SEF as soon as the Israeli law enables them—meaning at their early 30s –to increase the procedure’s potential success, and due to more traditional ideals of the life course in which women are expected to be married and start having (multiple number of) children at younger ages (Inhorn et al., 2020). Those interviewees further related to the stigmatization of “late” singlehood—which within the Jewish religious society is defined as such at younger ages.

As such, some of our religious interviewees criticized the Israeli legal age limitation for the usage of SEF (30–41) which they identified as jeopardizing the procedure’s success rates. This criticism uncovers the normative nature of this legal time frame, which can be interpreted as resting upon social ideas concerning the “right” time or timing for reproduction. This is especially the case since from a biological perspective, the “right” time for reproduction arrives at much younger maternal ages than 30–41. A few of our religious interviewees stressed how in their social sector, this time-frame is “too late”—uncovering intra-cultural differences in the ideals of collective timetables and biographical schedules.

When talking about social freezing, this group of interviewees further presented a unique idea of long-term family planning by stressing that for them, the decision to freeze eggs relies not simply on the wish to have a child, but on the wish to have as many children as possible, as it was put by a Jewish-religious interviewee:

“If I am getting married at the age of 35, so a secular woman getting married in that age she will have 2–3 children, (…) it is enough for her. For a religious woman it is too little. (…) When you look at it from the perspective of the religious public, this is something that is done by religious women who wants many children but (…) got married in a late age. (…). Fertility preservation (…) is not for being a mother in general, it is for being a mother of five”.

Following this line of reasoning, they plan on getting married and have their first children in a natural way, but then, at later ages, when already facing fertility decline, they will be able to use the frozen oocytes for continuing to bring more children. This type of long-term planning further highlights a unique variation of the perception of the usage of SEF as a responsible act of taking control over (future) fertility. It therefore seems that the usage of SEF among religious women represents an ongoing negotiation between medical calculation on the one hand and religious belief systems and traditional social norms on the other (Kılıç & Göçmen, 2018).

Similarly, to their secular counterparts, Jewish religious interviewees undertook SEF due to their inability to find a proper partner. However, this group represents a unique motivation while clearly relating to traditional family model and large family norms in which motherhood is a central feature of religious self-identity. It therefore seems that for these women, the usage of SEF represents a future oriented attempt directed at extending their reproductive timeline (Inhorn et al., 2020) or horizon.

Relating to the concept of ‘futurism’, Tavory and Eliasoph (2013), present a useful approach for making sense of the multiple kinds of future orientation which can be useful for our analysis. In their approach, they distinguish between ‘protentions’—an individual moment-by-moment anticipation of the future, ‘trajectories’ through time that involve certain long-term narratives and future oriented projects and goals, and ‘plans and temporal landscapes’ which include overarching temporal orientations towards the future that are often naturalized, taken for granted, and experienced as inevitable. All of our interviewees, reflecting all three types of motivations, use SEF as an attempt to synchronize and/or disentangle different trajectories (e.g., the trajectory of finding a partner and the trajectory of having children) (for relevant discussion see: Brown & Patrick, 2018). However, Jewish religious women’s reproductive temporalities also adhere to a very particular and distinctive reproductive plan and temporal landscape- reflecting naturalized and traditional future oriented collective temporal order.

This notion of collective temporality can be further discussed as representing a very transparent form of ‘reproductive futurism’ (Edelman, 2004). It is a basic temporal directedness of a social collective towards the future and future generations (Rimon-Zarfaty & Schweda, 2019). The concept of ‘reproductive futurism’ was also identified within the debate on ‘queer temporalities’ as a time line which adheres to heteronormative conceptions of a couple-oriented and reproductive futurity (Edelman, 2004; Lahad, 2012). The highly political context of the Jewish-Israeli pro-natalism with its demographic goals as well as underlying message of Jewish religious commandment to be “fruitful and multiply” (Donath, 2015; Kahn, 2000; Kanaaneh, 2002) frames this collective temporal notion. Within this context, Birenbaum-Carmeli et al. (2021), identified Jewish-women’s usage of SEF as reflecting their commitment to the “Jewish maternal imperative” (p. 346).

### Temporal motivations: cross-cultural perspective

Our case study reveals three main types of temporal motivations for using SEF, along national cultures and personal experiences. The first type, characterizing mainly of German SEF users, is aimed postponing reproduction and the need to make reproductive decisions. The second type is shared by both Israeli (secular) and German users. These women bank their eggs to buy more time in the hope that they will find the partner for whom they are waiting. Within this type of motivation, one can find cross-cultural differences at the cultural level of perception and argumentation (e.g., around the understanding of motherhood as (un)conditional, also reflected in the Israeli possibility to freeze embryos, as well as ideals of appropriate timing as reflected in the German ambivalence around “late motherhood”), but not in the personal context of decision-making. The third type which characterizes Israeli Jewish-religious users, reflects long-term family planning. Our case study therefore uncovers temporality formations embedded in gender and reproductive moral values, including the ‘extended present’, ‘waiting’, and ‘reproductive futurism’, respectively.

One can identify similarities between the women presenting the three types of motivations in terms of their personal (singlehood) status and biographical narratives. However, at the same time, the analysis of those motivations uncovers different temporality formations and logic. While the second type demonstrates the extent to which the phenomenology of singlehood produces a certain temporal identity that exceeds cultural boundaries, the other two represent a cultural contrast. Relevant cultural scripts to explain these differences include the high social expectations anticipated by German mothers (Fagnani, 2002; Mckay, 2011), and the Israeli pro-natalism which generally speaking frames the favorable Israeli approach to fertility medicine (Gooldin, 2008; Kahn, 2000), and preservation (Birenbaum-Carmeli, 2016b; Inhorn et al., 2020; Shkedi-Rafid & Hashiloni-Dolev, 2011; Rimon-Zarfaty et al., 2021). Further possible explanations may include the general promotion of individualism in Germany (Raz & Schicktanz, 2016), where familism and individualism are perceived as contradictory and the increasing value of individual self-realization was identified as leading to a decline in birth rates (Hashiloni-Dolev & Shkedi, 2007; Keller et al., 2005). This was also apparent in data indicating increased rates of childless (or childfree) women (DESTATIS, 2019). By contrast, the Israeli culture has been identified as a combination of individualism and collectivism reflecting the importance of family ties and genetic kinship (Lavee & Katz, 2003; Raz & Schicktanz, 2016).

## Conclusion

In our study, we examined how temporality is experienced and takes shape across societies in the particular context of cryo-fertility. Following our discussion about the connection between biology and temporality in the context of artificial cold (cryo), we therefore focused on SEF as a site for examining emerging temporalities and the corresponding experiences and attitudes. Our case-study however, has some limitation, mainly due to a limited sample size and focus on two particular socio-cultural contexts (Israel and Germany). Though our ability to generalize from the study is therefore limited, the cross-cultural perspectives nevertheless enabled us to reflect on how such temporalities can vary and are influenced by socio-cultural factors. These factors include normative cultural perceptions regarding reproductivity as a part of the self-image and gendered perceptions regarding the ideal life-course and its stages. The usage of the term ‘reproductive temporalities’ therefore goes beyond biological halting to socio-cultural understandings of reproductive time and timing (Smith-Hefner & Inhorn, 2020). In other words, our case study highlights both intra- and cross-cultural differences in reproductive temporalities. Thus, the abstract-individualistic outlook dominating the philosophical and bioethical perspectives on cryobiology is expanded by including the particular socio-cultural and political contexts.

Our case study also wants to advance the understanding of cryobiology in the broader sense (for relevant discussion see: Lemke, 2019). For this, we wish to go back to the concept of ’cryopolitics‘ as a general theoretical reference frame. While artificial cold (cryo) has been recognized and analyzed as a biopolitical tool (Radin & Kowal, 2017), our cross-cultural comparative findings uncover and allow reflection on cryopolitical mechanisms and the different ways in which those may operate in different social contexts. Our case study demonstrates how different temporalities and time regimes are entangled with different socio-cultural and socio-political framework conditions. Drawing on Oikkonen (2020) observations in the context of DNA research, cryopreservation or in our case cryo-fertility technologies are not epistemically nor politically innocent. Those become particularly apparent by the investigation of the ways such cryo-technologies develop, are invoked and negotiated in culture as well as the uncovering of particular ways of perceiving and negotiating temporality. By gaining empirical insights into the different ways in which frozen materials become located in specific normative temporalities (Kroløkke, 2019, p. 531), our case study extends our understanding of the concept of ‘cryopolitics’ and its particular expressions (Lemke, 2019). Our case study therefore, highlights the theoretical implications and importance of gendered and cultural imaginaries (re)constructing medical technological innovations and related temporalities.

‘Cryopolitics’ can hence serve as an important framework for understanding, analyzing, and theorizing the ways in which nowadays women’s lives, bodies, and reproduction are disciplined and regulated to fit dominant norms and social expectations (Kroløkke et al., 2020) and the ways those are internalized, negotiated, and challenged by women. Such cultural imperatives include, as our case study demonstrates, culturally-idealized (reproductive) temporalities and movement through the life course (Brown & Patrick, 2018); perceptions and understandings of emerging technologies and the extent to which the use of such technologies for reshaping one’s movement through life is viewed as legitimate and acceptable (ibid, p. 977); and finally, the different ways in which those are linked to cultural scripts and anxieties (ibid) in turn facilitating the effort to choreograph ontological state (e.g. that of ‘mother’ or ‘family’) (Radin & Kowal, 2017, p.13).

## Data Availability

Not applicable.

## Footnotes

1 Cryoprotectants are agents (i.e. chemical compounds such as glycerol, ethylene glycol etc.) used to prevent ice formation and damage to cells and tissues during cryopreservation by increasing the concentration of the solutes (Kar et. al., 2019).

2 In 1986, the first birth from cryopreserved eggs (using a slow freezing method) was reported. The first pregnancy and birth from vitrified oocytes were reported in 1999 (Gook, 2011).

3 Apart from the crystallization of the oocytes, the second challenge faced by scientists was the difficulty in achieving fertilization due to zona pellucida hardening caused by the cryopreservation process. This challenged has been circumvented by the development of ICSI (intracytoplasmic sperm injection) which improved the fertilization rates of previously frozen eggs (Baldwin et al., 2014; Wang & Sauer, 2006).

4 In Israel interviewees age span was between 32 and 47 with an average age of 36.1 (34.4 at the time of SEF usage). Of these, 15 were single, 4 divorced and 4 married (though single when using SEF). In Germany, interviewees’ age span was between 29 and 45 with an average age of 34.6 (33.3 at the time of SEF usage), of whom 12 of the interviewees were single, 3 divorced and 1 married (though single when using SEF).

5 IRB approvals were obtained from the University Medical Center Goettingen (Germany) [reference no. 22/1/18] and Ben Gurion University of the Negev (Israel) [reference no. 1571–2]. Interviewees were provided with detailed information forms concerning the research and signed an informed consent form. The procedure and the forms were approved by both IRBs.

6 Due to the qualitative nature of our research, in presenting the different motivations, we provide the following indicational quantifiers: *minority*- indicating approximately 15%-20% of the interviewees; *majority/many/most*- indicating 65%-70% of the interviewees or more; *few*- indicating 10% or less; and *some*- indicating about 40%-50% of the interviewees.

7 Interestingly, the controversy around late motherhood is also apparent when analysing German academic publications on the issue of SEF (e.g., Bernstein and Wiesemann, 2014; Bittner and Eichinger, 2010; Bozzaro, 2018; Weber-Guskar, 2018). Such publications relate (also from a critical perspective) to the argumentation that motivations such as postponing childbirth in favour of self-realization or due to the inability to make fundamental life decisions (and SEF which enables that) are problematic- highlighting implicit normative expectations regarding reproductive timing and its proper location in the life course.

8 The permissibility of SEF and its legitimacy by some rabbinical authorities can be further analyzed within the context of the halachic idea of family integrity and the halachic problem with sperm donation (Birenbaum-Carmeli, 2016a; Inhorn et al., 2020; Schenker, 2008).
